# Impact of heavy load activity on cardiovascular system: echocardiographic assessment of informal construction workers heart in Cameroon

**DOI:** 10.11604/pamj.2014.17.79.3674

**Published:** 2014-01-31

**Authors:** Francis Nde, Jules Nebo, William Ngatchou, Carine Tchatchoua, Albert Mouelle Sone, Christophe De Brouwer

**Affiliations:** 1 Centre de Recherche Santé environnementale & Santé au travail, École de Santé Publique, Université libre de Bruxelles, Bruxelles, Belgique; 2 Polyclinique de Bonaberi, Douala, Cameroun; 3 Université libre de Bruxelles 808, Hôpital Saint Pierre, Bruxelles, Belgique; 4 Faculté de Médecine et des Sciences Pharmaceutiques de l'Université de Douala, Cameroun

**Keywords:** Cardiac change, workers heart, left ventricle, septum, posterior wall thickness, Work load, impact of physical activity

## Abstract

**Introduction:**

Physiological cardiac hypertrophy and dilation are common findings in heavy physical load activity. We carried out this study to investigate the relationship between construction work and cardiac parameters adaptations, by comparing healthy masons to office workers on heart ultrasound.

**Methods:**

The study was carried out on, 50 construction workers and 50 office workers matched for their weight, height and age. Systolic and Diastolic blood pressures, Left Ventricular diameter and thickness, Septum wall thickness and Left ventricular mass index were measured and calculated

**Results:**

Heart rate, systolic and diastolic blood pressures were lower in construction workers, as compared to office workers: respectively 63±7 bpm vs. 75±6 bpm (p = 0.000); 120.1±7 mmHg vs. 130.5±9 mmHg (p = 0.000) and 68.5±7 mmHg vs. 77.0 ±9 mmHg (p = 0.000). Construction workers had a thicker septum and posterior wall: respectively 10.3 ± 1.1 mm vs. 8.9 ± 0.9 mm (p = 0.000); and 9.0 ± 1.2 mm vs. 8.1 ± 0.8 mm (p = 0.000).

**Conclusion:**

Conclusion We deducted that heavy load work has an impact on the heart mensuration. The past occupational history has to be taken into consideration during initial medical assessing of a worker in for a new job so as to avoid erroneous conclusions.

## Introduction

Many studies showed the role of professional activities or environmental condition on cardiovascular system [1]. Workers will therefore be affected on different manners by their work [2]. Cardiovascular diseases related to occupational stress is also scientifically known [3]. Scientists have been interested in echocardiographic and cardiovascular variations due to exposure to lead [4–8]. William Ogle [6] observed the first, in the years eighties the effect of heavy work on heart. To our awareness, no study has been done to assess relationship between cardiac modifications seen on ultrasound in construction workers in developing countries, particularly in Cameroun. In Cameroon, occupational medicine is still on an embryonically status. A lot is being done on juridical aspect, thru some steps tones taken, but at the moment, workers are not well followed up and there are only few qualified occupational physicians for millions of official workers. Informal workers are not followed in regards to safety and health despite the fact that they are all exposed to hazards and environmental health risks. Moreover, they are more prone to health and safety hitches due to the fact that they are mainly less cultured and educated regarding the risks at work settings. We examined 50 health workers, all male waged in Douala municipality in Cameroon, occupied in construction informal activity, and compared them with 50 health office workers matched for gender and age. We carried out this study to find out if there was a physical and physiological cardiac alteration on echography observed in construction workers as compared to office workers that could be due or attributed to their occupation.

## Methods

This research is a prospective cohort study designed to measure the dimensions of heart on ultrasound on 50 masons and compare to 50 office workers. The masons were recruited form different part of Douala town in Cameroon. Office workers were recruited form different companies. In all cases, they were randomly selected. We tried to match to two groups to reduce biases that could be due to weight, height and age. None of the subjects were exposed to sport activity. We obtained an informed consent from all the subjects participating to this study. The Cameroon Society of General Medicine approved our protocol. After approving the inform consent form, a health questionnaire was filled by each worker, with general information including age, weight and height. This was followed by a clinical examination with two consecutive measurement with a 5 min interval time between the two measurements of blood pressure at rest at the left upper arm, then cardiac and lung auscultation. Finally, heart ultrasound was performed by one ultrasound qualified operator, on a patient at a 30 degree left lateral position. For this study, we used an ultrasound from Siemens. Septum and posterior wall thickness were measured, followed by ventricular cavities measurements: left ventricle and atrium. The left ventricular relative mass was calculated using the formula All subjects with past medical history of cardiovascular diseases, regular sportive activity and ongoing cardiovascular treatment were excluded from our sample. The analyses were performed using IBM SPSS Statistics 20. From initial data recorded, some calculations were done. Devereux formula was used to calculate left ventricular mass. Dubois formula was used to calculate body surface. Reichek formula was used to calculate diastolic relative wall thickness. We used descriptive statistics median, minimum and maximum and non-parametric Mann-Whitney t tests for unmatched samples to compare cardiac ultrasound parameters between Construction and office workers. We calculated the Spearman correlation parameter to measure the correlation between continuous variables in the two categories of workers.

## Results

Our two groups were comparable in terms of average age, weight, height, budy mass index and budy surface areas (Table 1, Table 2). All possible differences could therefore be related to other explanation than just anthropological differences. The analysis indicated that there was a significant lower heart rate, Systolic and diastolic blood pressure in construction workers at rest as compared to office workers, but all remaining within the normal ranges, showing no pathological values.

**Table 1:** General and clinical parameters

Parameters	Office workers (n = 50)	Construction workers (n = 50)	P value
	Mean (sd)	Mean (sd)	
Age(years)	36.1 (9.1)	37.4 (8.2)	NS
Weight (kg)	74.6 (5.7)	74.9 (4.8)	NS
Height (cm)	175.1 (6.5)	174.3 (5.0)	NS
Heart rate (b/min)	75.3 (5.8)	63.6 (5.1)	0.000
Systolic Blood Pressure	130.5 (9.2)	112.1 (8.6)	0.000
Diastolic Blood Pressure	77.3 (8.6)	68.5 (7.0)	0.000
Budy Surface Area	1.90 (0.08)	1.90 (0.08)	NS
Budy Mass Index	24.61 (2.2)	24.50 (2.1)	NS

**Table 2:** Echocardiographic parameters

Parameters	Office workers	Construction workers	P value
	Mean (sd)	Mean (sd)	
Left Atrium Diameter (mm)	35.1 (1.5)	39.9 (2.2)	0.000
Left Ventricular End Diastolic Diameter (mm)	50.3 (2,3)	53.2 (1.8)	0.000
Meridional Wall Stress (dyn/cm^2^)	41.6 (1.7)	40.9 (2.3)	NS
Left Ventricular End Systolic Diameter (mm)	30.2 (4.6)	31.7 (3.9)	NS
Posterior Wall Thickness (mm)	8.0 (0.8)	9.0 (1.2)	0.000
Septum Wall Thickness (mm)	8.9 (0.9)	10.3 (1.1)	0.000
Sum of Ventricular Wall Thickness (mm)	16.9 (1.3)	19.3 (1.7)	0.000
RWT (h/R)	0.336 (0.031)	0.364 (0.037)	0.000
Left Ventricular Mass Index (g/m^2^)	90.4 (11.9)	120.2 (15.5)	0.000
Ejection Time (milisec)	30.5 (1.8)	28.8 (2.3)	NS
DT_E (milisec)	147.0 (5.1)	145.9 (3.9)	0.000
E Wave	79.1 (3.3)	80.0 (3.1)	NS
A Wave	53.2 (3.1)	52.4 (2.9)	NS
E/A Ratio	1.49 (0.10)	1.53 (0.10)	NS
Shortening Fraction	0.40 (0.10)	0.40 (0.07)	NS

The analysis indicated that there were larger left atrium diameter and left ventricular end diastolic diameter, thicker septum and posterior wall, as well as bigger sum of ventricular wall thickness and left ventricular mass index. All these differences were statistically significant (p < 0.001). Ejection time was longer in office workers, but the difference was not statistically significant, showing a physiological changes rather than a pathological one. Similarly, there were no statistical significance in the differences observed between the two groups in regards to DT_E, E wave, A wave and E/A ratio as well as shortening fraction, once more showing the physiological variation observed.

**Figure 1:**
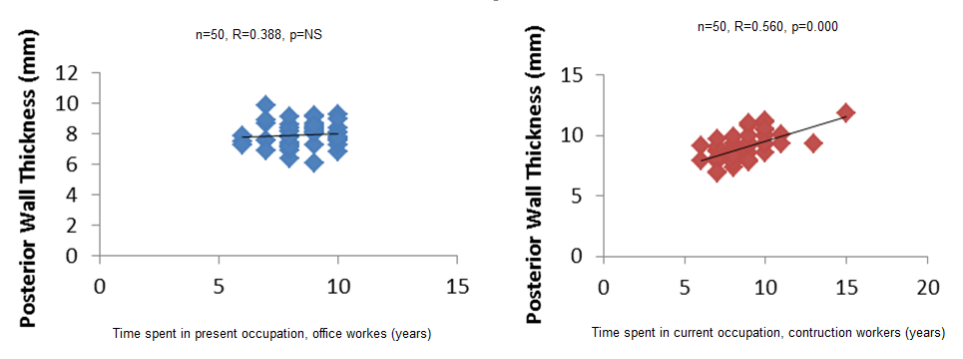
Correlation between time spent at activity and posterior wall thickness

**Figure 2:**
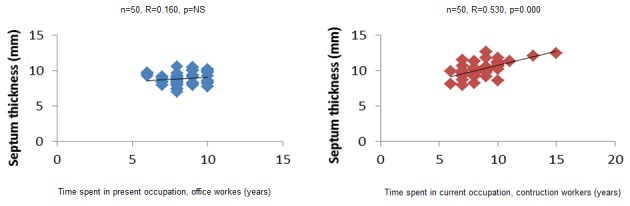
Correlation between time spent at activity and septum thickness

**Figure 3:**
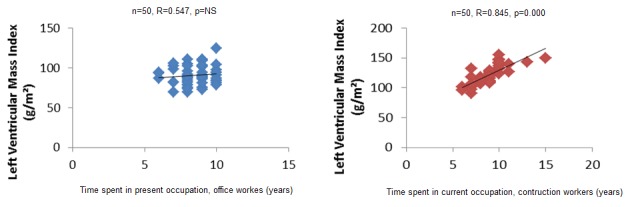
Correlation between time spent at activity and left ventricular mass

Non parametric Spearman correlation shows a correlation between wall thickness and left ventricular mass index, and time spent at function in years, in construction workers (Figure 1, Figure 2, Figure 3). In office workers, this correlation is not observed. In other words, the more one could spend practicing the activity, the thicker the posterior wall, septum and left ventricular mass in construction workers. These variation, though observed in office workers, were lower. This argument also goes in the sense of heaviness of physical load in construction workers as compared to office workers, however, these changes were still in the normal range. No pathological value was observed in construction workers.

## Discussion

The major finding of this study in Cameroonian cohort is that constructors workers had had higher LV mass as compare to office workers. Echographically measured left ventricular mass is an important predictor of adverse events in the general population [7]. LV mass is associated with multiple factors including age, blood pressure, BMI, and sex [8–11]. Regular physical exercise is also associated with LV mass increase [12]. Few studies has been done to understand the physical strain impose to constructions workers. Despite some limitations, these studies showed that physical demands of that construction workforce is high and the complications on health are obvious [13], since the physiological threshold recommended is routinely exceed.

In the present study, the fact that construction workers had lower heart rhythm and thicker ventricle wall is probably related to physical heaviness of activities needed in this profession. The same situation had been described in trained athletes and non-athletics population practicing regular exercise. Resistance training is associated with proportional increase in the left ventricular wall thickness [14, 15]. The modality, intensity, duration and frequency of exercise as well as body size, gender and genetics determinants influence the grad of cardiac adaptations [16]. Indeed in this study, a positive correlation between time spent in the job by construction workers and LV mass, LV Thickness, Post wall thickness shows that the longer they stayed at job, the stronger the association. In the literature it is shown that the highly trained endurance athletes show more enlarged heart [17]. Our construction workers could be assimilated to highly trained athletes in regards to heaviness of their activity.

## Conclusion

The increase in parameters found on echography in construction workers may be related to their heavy work activity. The suggestion is made by the fact that factors that could cause a bias in the conclusion such as smoking habit and sport are controlled. We suggest that special attention be taken in regards to assessment of construction workers as they may be at risk of cardiovascular complications as compare to other professionals.
